# Penile Rehabilitation Therapy with PDE-V Inhibitors Following Radical Prostatectomy: Proceed with Caution

**DOI:** 10.1155/2009/852437

**Published:** 2009-01-25

**Authors:** M. Eric Brewer, Edward D. Kim

**Affiliations:** Graduate School of Medicine, The University of Tennessee, Knoxville, TN 37920, USA

## Abstract

Penile rehabilitation therapy following radical prostatectomy is a much debated topic. Erectile dysfunction is still a significant contributor to postoperative morbidity following radical prostatectomy, despite meticulous nerve-sparing technique. Secondary smooth muscle changes in the penis have been identified as the underlying causes of penile atrophy, veno-occlusive dysfunction, and fibrosis. Initial observations that intracavernous injection therapies used on a regular basis postoperatively resulted in improvements in the return of spontaneous erectile function led to the development of penile rehabilitation protocols. Chronic dosing of PDE-V inhibitors is now commonly used by urologists after radical prostatectomy. Despite the current enthusiasm of penile rehabilitation therapy, current scientific evidence with clinical trials is still limited.

## 1. Introduction

Erectile dysfunction (ED) continues to be a significant complaint among men undergoing radical prostatectomy (RP), despite meticulous nerve-sparing technique. The discrepancy between preservation of the nerves and recovery of erectile function leads us to believe that the etiology is likely multifactorial. Smooth muscle alterations and fibrotic changes in the penis following surgery were identified as underlying causes of penile atrophy, veno-occlusive dysfunction, and Peyronie's-like changes. Other mechanisms, such as penile hypoxia, are likely to contribute to post-RP erectile dysfunction as well.

The topic of penile rehabilitation therapy (PRT) has become an area of intense interest over the last decade. Initial observations that intracavernous injection therapies used on a regular basis postoperatively resulted in improvements in the return of spontaneous erectile function led to the development of penile rehabilitation protocols [1]. A central question is whether vasoactive therapies, such as oral type V phosphodiesterase (PDE-V) inhibitors and intracavernous or intraurethral alprostadil, can lessen or reverse the effects of causative factors for ED. As several recent articles in Advances in Urology have thoroughly reviewed the benefits of PRT, the purpose of this article is to provide caution to the present enthusiasm for penile rehabilitation.

## 2. Penile Injury Following RP: Basic Science

The basic science behind the mechanism of erection has been discussed previously and is beyond the scope of this review [2]. With that said, it is important that we understand the underlying hypothesis of penile rehabilitation therapy. In the 1990s, it was recognized that denervation injury to the penis affects the cavernous smooth muscle. This is similar to the skeletal muscle atrophy that is seen following spinal cord injury. Using a rat model, Klein et al. were the first to demonstrate that denervation of the penis leads to apoptosis [3]. Then in 2003, User and McVary were able to show penile apoptosis as early as 1 day after cavernous nerve ablation in a rat model [4]. This apoptotic process is directly related to atrophy and fibrosis. The hypothesis is that PDE-V inhibitors promote penile rehabilitation by stimulating smooth muscle cell replacement via a cGMP mechanism and reducing collagen synthesis via phosphokinase G activation [5]. By performing percutaneous penile biopsies at the time of RP and 6 months later, Schwartz et al. were the first to demonstrate that early use of 100 mg of sildenafil after RP may preserve intracorporeal smooth muscle content [6]. Interestingly, those taking 50 mg of sildenafil under the same dosing regimen showed no statistically significant change in smooth muscle content. There was no control group in this study and only 21 of the enrolled 40 men were available for follow-up. The effects on the long-term return of erectile function were not determined. Rajfer et al. from UCLA demonstrated in several studies that rats treated with PDE-V inhibitors had no significant increase in the penile shaft collagen content [5, 7, 8]. These studies have provided important animal model documentation of the benefit of PDE-V inhibitor therapy for the prevention of functional and histologic changes in the penis that can occur after nerve damage.

## 3. Limitations of Present Clinical Studies

While there have been many studies in the rat model showing the benefits of local vasoactive therapies, the crossover to clinical significance in the human has been more difficult to prove (see Table 1). Montorsi et al. showed in 1997 that men who performed penile injections had a superior rate of return of adequate erections following RP than men who did not do injections [1]. However, the study did not include preoperative parameters of erectile function or the use of a validated questionnaire. Also, the small number of patients included in the study decreases its power. Additionally, the short duration of follow-up of 12 weeks limits any conclusions regarding long-term impact of therapy. Nevertheless, this study was the first clinical report to suggest a benefit of a penile rehabilitation strategy using a pharmacologic therapy.

The evidence for the use of oral agents for penile rehabilitation is even weaker. In 2005, Mulhall et al. reported in a nonrandomized study that men who underwent pharmacologic penile rehabilitation following RP resulted in higher rates of spontaneous functional erections [9]. Men who had fully functional erections by self-report preoperatively were encouraged to participate in a penile rehabilitation program for at least 12 months following surgery. Patients who decided to participate constituted the rehabilitation group, while those who decided not to participate but presented for follow-up for periodic evaluation of their erectile function constituted the nonrehabilitation group. There were 58 men in the rehabilitation group and 74 in the nonrehabilitation group, but no placebo arm. This study had a strong patient selection bias, as patients were allowed to select treatment or observation. In addition, only men who completed 18 months of therapy were included, meaning that men who dropped out due to lack of efficacy were not part of the analysis.

The benefit induced by a PDE-V inhibitor rehabilitation approach compared to an on demand PDE-V administration has not been confirmed. In a follow-up to their first study [1], Montorsi et al. prospectively studied a cohort of 80 patients submitted to bilateral NSRRP with adequate erectile function data 12 months after surgery, as presented in their abstract at the 2006 annual meeting of the American Urological Association [10]. Patients were assigned to four different groups: no erectile therapy, intracavernosal injection of prostaglandin E-1 on demand, PDE-V inhibitor on demand, and PDE-V inhibitor therapy either daily or every other day for 3 months. No significant difference was found in the mean IIEF score between patients treated with daily therapy versus on demand therapy.

Bannowsky et al. from Germany reported in 2008 their findings of low-dose sildenafil for rehabilitating erectile function after nerve-sparing RP [11]. Twenty-three men with preserved erectile function based on Rigiscan testing 1-2 weeks postoperatively received sildenafil 25 mg nightly versus 18 men who received placebo for 52 weeks. There was a significant difference in IIEF-5 score and time of recovery of erectile function between the groups (*P* < .001), with potency rates of 86% versus 66%. The authors concluded that in cases of early penile erection, daily low-dose sildenafil leads to a significant improvement in the recovery of erectile function. However, the limitations to this study are that it was performed at a single center and 95% of patients had nocturnal erections following catheter removal, which is not typical.

Padma-Nathan et al. performed the first randomized, placebo-controlled trial of chronic PDE-V inhibitor therapy for the purposes of penile rehabilitation and presented their findings at the 2003 American Urological Association annual meeting [12]. They concluded that nightly administration of sildenafil for 9 months following nerve-sparing radical retropubic prostatectomy (NSRRP) increased the return of spontaneous erections compared with placebo. However, this study has been criticized for the seemingly low percentage (4%) of men considered responders in the placebo arm. Additionally, the criteria for being considered a responder were stringent, as responders were defined as those having a combined score of >8 for IIEF Q3 and 4 and positive response to the question “Over the past 4 weeks, have your erections been good enough for satisfactory sexual activity?” [13] The findings of this study were recently published in the International Journal of Impotence Research [14].

A subset of these men had nocturnal penile tumescence and rigidity (NPTR) testing performed preoperatively and at various time points postoperatively [15]. The authors concluded that nightly sildenafil objectively improved nocturnal erections versus placebo.

The limitation of this study is that while being suggestive, nocturnal tumescence data interpretation does not necessarily correlate with return of clinically usable erections.

In contrast, in the largest randomized, double-blind, double-dummy, multicenter, parallel study done to date, Montorsi et al. recently reported on a vardenafil trial after bilateral nerve-sparing RP [16]. A total of 628 men were randomized to placebo, nightly vardenafil, or on demand vardenafil for 9 months, followed by a 2-month washout period, and an optional 2-month open-label period. The results clearly show that nightly dosing with vardenafil did not have any effect beyond that of on demand use. Following washout, IIEF-EF scores ≥22 were achieved in 28.9%, 24.1%, and 29.1% of patients for placebo, vardenafil nightly, and vardenafil on demand groups, respectively, showing no statistically significant difference among the groups. Mean per-patient success rates for intercourse completion were not significantly different among treatment groups either. While on demand dosing was efficacious, nightly vardenafil for the purpose of penile rehabilitation was not efficacious. This well-designed study provides a cautionary note for the present enthusiasm of oral PDE-V inhibitors for penile rehabilitation therapy. The authors concluded that: “the finding that on demand dosing is more effective in improving both erectile function and sexual intercourse completion rates within this patient population prompts reconsideration of the current practice of prescribing nightly PDE5 inhibitor therapy, as on demand use of vardenafil if equally effective in men with ED following NSRP.”

Point-counterpoint debates have become common regarding the efficacy of penile rehabilitation following RP. In one such example, Abraham Morgentaler from Harvard University listed several intriguing arguments [17]. First, he questioned the theory that penile rehabilitation helps to reverse the chronic hypoxia and ischemia of the flaccid penis following RP. “But why is there reason to suspect that the flaccid penis is hypoxic, despite having venous oxygen tension? After all, the endothelium of all venous structures suffers no ill effects despite a lifetime of exposure to oxygen levels that are well below those seen in arterial blood. Second, there is no reason to believe that the penis is ischemic following RP. If it were ischemic, would it not eventually become necrotic? Furthermore, PDE-V inhibitors do not increase blood flow to the flaccid penis, so how can benefit occur?”

Similarly, in a more recent point-counterpoint debate at the 2008 AUA Annual Meeting in Orlando, Fla, Craig Donatucci from Duke University was charged with providing the contrary argument as whether penile rehabilitation was effective after RP. He presented many of the same points listed above, describing the pitfalls of the clinical trials that have been presented thus far. He stated: “There is not yet enough evidence to declare penile rehabilitation effective; but I am not sufficiently convinced of the ineffectiveness of penile rehabilitation to recommend against it. My opinion is that the benefits of penile rehabilitation are “not proven”; yet it is currently the standard of care” [18].

## 4. Conclusions

The study of penile rehabilitation is in active evolution. There are intriguing data supporting the use of penile rehabilitation in the animal model, but these studies may not translate into human effect. While many published reports of clinical trials point toward improved outcomes with active treatment, these studies have significant limitations. Most recently, the only large, randomized, placebo-controlled trial to date showed no clear benefit of rehabilitation with vardenafil versus on demand dosing. In fact, quite the opposite was shown. Clearly, more randomized, placebo-controlled trials are needed. Unfortunately, these present many challenges. First, these trials prove costly with poor long-term compliance and significant early dropout rates. Second, it cannot be overlooked that differences in nerve-sparing technique and outcomes between surgeons can confound interpretation of results. Even though the benefits of penile rehabilitation are still questionable, the practice of frequent and chronic administration of PDE-V inhibitors has become standard of care for many urologists. We suggest proceeding with caution when encouraging our patients to adhere to a rehabilitation regimen, until more definitive results are available.

## Figures and Tables

**Table 1:** Review of clinical studies.

Authors	*n* (number of patients)	Conclusions and *limitations*
Montorsi et al. (1997)	30	Alprostadil injections led to superior rate of return of adequate erections; *no preoperative EF parameters, no questionnaire, small number of patients, short follow-up*

Mulhall et al. (2005)	132	Pharmacologic penile rehabilitation protocol results in higher rates of spontaneous erections and erectogenic drug response; *no placebo arm, strong patient selection bias, dropouts were not included*

Montorsi et al. (2006)	80	No significant difference is IIEF scores of patients using on demand versus daily PDE5 inhibitors post-NSRRP; *compliance not reported*

Bannowsky et al. (2008)	43	Daily low-dose sildenafil leads to improved recovery of EF post-NSRRP (86% versus 66% placebo); *small number of patients, not placebo-controlled, single center, only included patients who showed preserved EF post-operative with Rigiscan testing*

Padma-Nathan et al. (2008)	76	First placebo-controlled trial suggesting benefit of daily PDE5 inhibitor use: nightly sildenafil increased return of spontaneous erections; *low percentage (4*%*) considered responders in placebo arm, but strict definition of responders*

McCullough et al. (2008)	54	Nightly sildenafil improved nocturnal erections versus placebo; *nocturnal tumescence data do not necessarily correlate with clinically usable erections*

Montorsi et al. (2008)	628	Nightly vardenafil did not have any effect beyond that of on demand use
